# Supplementary material for: Characterization of Mechanical and Cellular Effects of Rhythmic Vertical Vibrations on Adherent Cell Cultures
Source: Bioengineering (Basel). 2023 Jul 6;10(7):811. doi: 10.3390/bioengineering10070811 (PMC10376548; doi:10.3390/bioengineering10070811)
Supplement: Supplementary file 1 [file bioengineering-10-00811-s001.zip › figure_s1_manual_gating_EdU.pdf]

Manual gating of EdU flow vibrated HeLa cells

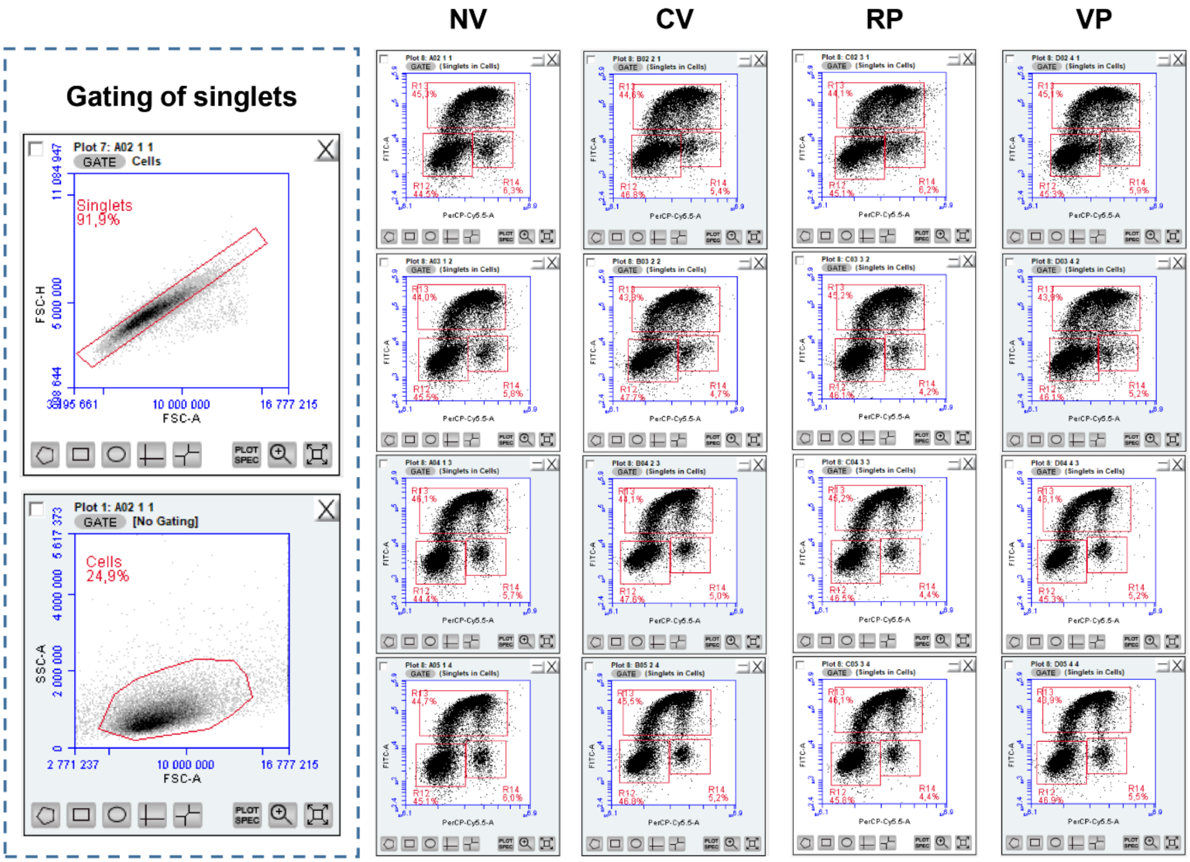

Supplementary Figure S1: Cell proliferation analysis using EdU proliferation kit
