# Supplementary material for: Characterization of Mechanical and Cellular Effects of Rhythmic Vertical Vibrations on Adherent Cell Cultures
Source: Bioengineering (Basel). 2023 Jul 6;10(7):811. doi: 10.3390/bioengineering10070811 (PMC10376548; doi:10.3390/bioengineering10070811)
Supplement: Supplementary file 1 [file bioengineering-10-00811-s001.zip › figure_s2_algorithm_test_blur_len_thick.pdf]

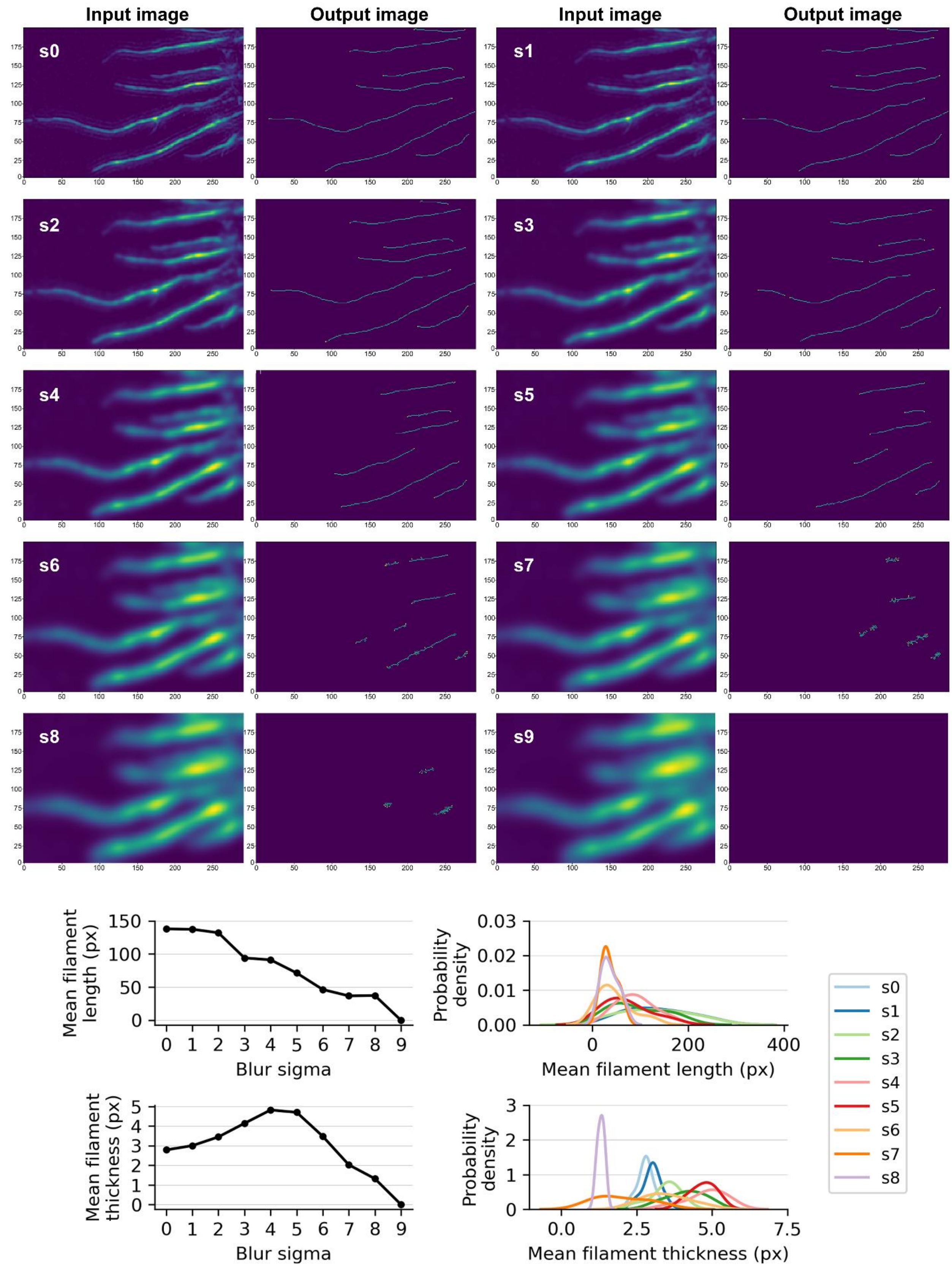
Input image
Output image
Input image
Output image
s0
s1
s2
s3
s4
s5
s6
s7
s8
s9
Mean filament length (px)
Blur sigma
Probability density
Mean filament length (px)
Mean filament thickness (px)
Blur sigma
Probability density
Mean filament thickness (px)
s0
s1
s2
s3
s4
s5
s6
s7
s8

Supplementary Figure S2: Algorithm test using gaussian blur filter of filament lengths and thicknesses

The results of the Gaussian blur test using different sigma (value determining the amount of blur) values in terms of filament lengths and thicknesses. The sigma values ranged from 0 to 9 (indicated as s0 to s9). S0 was the original image. S9 was the highest limit before the algorithm could not recognize any filament. As the sigma was increased, the number of pixel representations of the filaments reduced in the output images. Accordingly, the result of the mean filament length was stable up to s4 and started decreasing subsequently. The distribution stayed normal throughout the experiment. However, the width of the distribution narrowed because fewer filaments were identified, and shorter filaments were ``lost'' earlier than longer filaments from the original image. The mean thickness increased towards the middle of the sigma range but decreased after s6. One pixel represents ~0.148μm in these images.
