# Supplementary material for: Characterization of Mechanical and Cellular Effects of Rhythmic Vertical Vibrations on Adherent Cell Cultures
Source: Bioengineering (Basel). 2023 Jul 6;10(7):811. doi: 10.3390/bioengineering10070811 (PMC10376548; doi:10.3390/bioengineering10070811)
Supplement: Supplementary file 1 [file bioengineering-10-00811-s001.zip › figure_s3_algorithm_test_blur_ang.pdf]

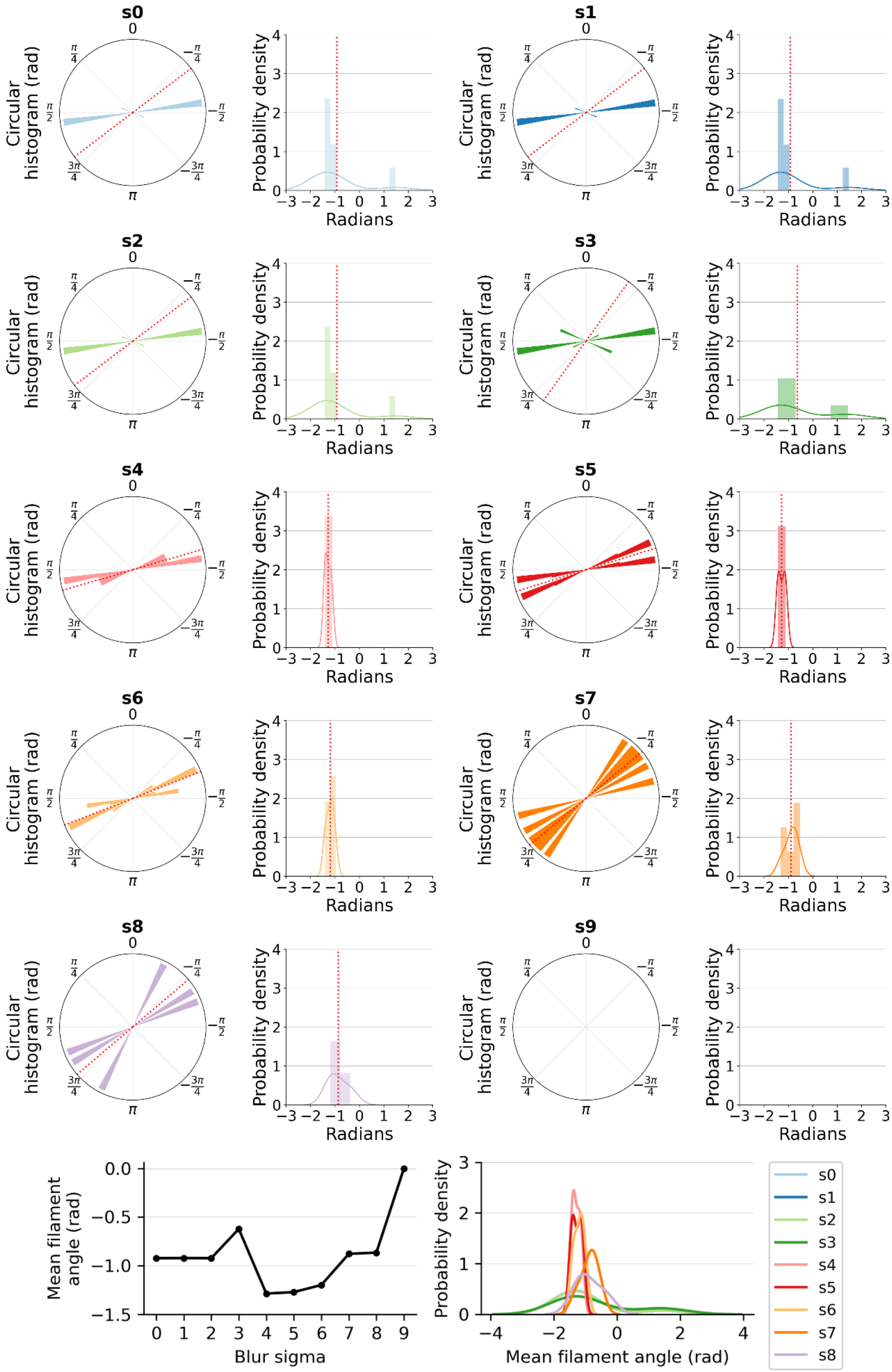

s0
s1
s2
s3
s4
s5
s6
s7
s8
s9
Circular histogram (rad)
Probability density
Radians
Mean filament angle (rad)
Blur sigma
Probability density
Mean filament angle (rad)
s0
s1
s2
s3
s4
s5
s6
s7
s8

**Supplementary Figure S3: Algorithm test using gaussian blur filter of filament angle**

The results of the blur test in terms of filament angles, not directions. The circular histogram (the matplotlib polar plot in Python) in radians helps to visualize the angles of the filament representations in the output images in Figure S4. The red-dotted lines indicate the mean in each result. The angle distribution shows the relative amount of filaments at a specific angle. As was the case with filament lengths and thicknesses, a decrease in the number of identified filaments from s4 affected the results negatively. Thus, similar to results shown in Figure S4, the width of the distribution becomes narrower from s4. However, the mean angle of the filaments altered more unpredictably than the mean length and thickness, although it remained generally steady until s3. As indicated in Figure S4, no filaments were identified in s9; therefore, no data was produced for that blur strength.
