# Supplementary material for: Characterization of Mechanical and Cellular Effects of Rhythmic Vertical Vibrations on Adherent Cell Cultures
Source: Bioengineering (Basel). 2023 Jul 6;10(7):811. doi: 10.3390/bioengineering10070811 (PMC10376548; doi:10.3390/bioengineering10070811)
Supplement: Supplementary file 1 [file bioengineering-10-00811-s001.zip › figure_s4_algorithm_test_image_overlay.pdf]

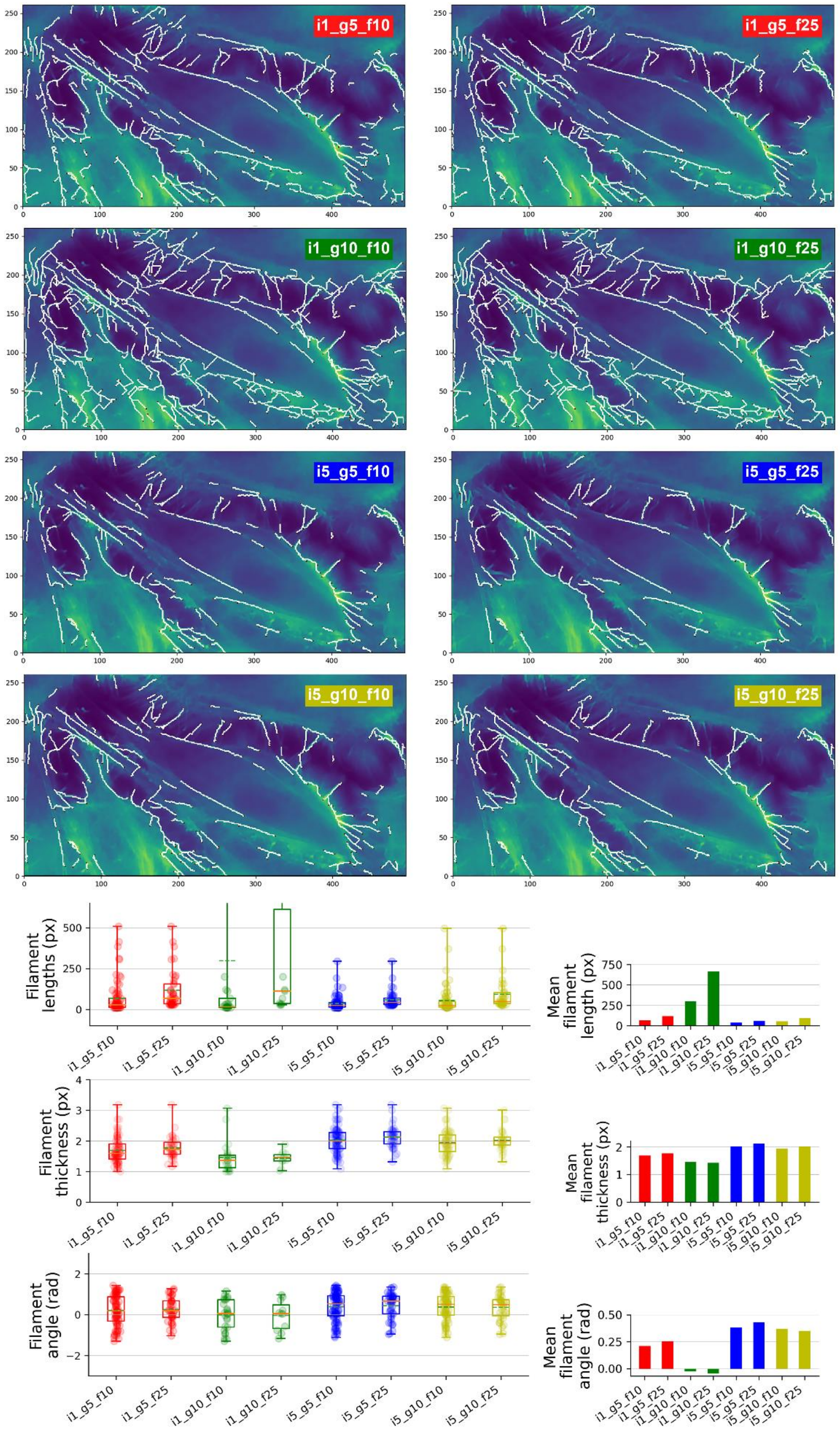

i1_g5_f10
i1_g5_f25
i1_g10_f10
i1_g10_f25
i5_g5_f10
i5_g5_f25
i5_g10_f10
i5_g10_f25
Filament lengths (px)
Mean filament length (px)
Filament thickness (px)
Mean filament thickness (px)
Filament angle (rad)
Mean filament angle (rad)

Supplementary Figure S4: Algorithm test comparing several algorithm parameters with extracted features overlaid on the original microscopic image

Superimposed pixel representations of the F-actin filaments on the original microscopic image and the results of the parameter test in terms of filament length, thickness, and angle. The box plots show the distribution of the data. The box represents the interquartile (the middle 50%) of the data points, the green dotted lines represent the mean, and the orange lines represent the median. The bar plots show the mean values of each parameter condition. The different color groups include a set of parameters where only one out of three was varied. For example, in the red group, IF and GF were kept the same while the FF was varied from 10 to 25.
